# Efficacy of Ozonated Gels and Oils As Adjunctive or Monotherapy in Periodontitis: A Systematic Review and Meta‐Analysis of Randomized Controlled Trials

**DOI:** 10.1002/cre2.70439

**Published:** 2026-08-25

**Authors:** Mahdi Bahrami, Mahsa Etemadi, Reyhaneh Abrishami, Farshad Alipour, Shiva Hadizadeh, Faezeh Jamshidian, Nooshin Abedinzadeh, Abolfazl Akbari, Edris Pordel

**Affiliations:** ^1^ Student Research Committee, Faculty of Dentistry Mashhad University of Medical Sciences Mashhad Iran; ^2^ Department of Periodontics, School of Dentistry Mashhad University of Medical Sciences Mashhad Iran; ^3^ Women Reproductive Health Research Center Tabriz University of Medical Sciences Tabriz Iran; ^4^ Department of Pediatric Dentistry, Dental School Sabzevar University of Medical Sciences Sabzevar Iran

**Keywords:** gel, meta‐analysis, oil, ozone therapy, periodontitis, randomized clinical trial

## Abstract

**Objectives:**

This study aimed to evaluate the available clinical evidence on the use of ozonated gels and oils in the management of periodontitis.

**Material and Methods:**

Embase, SCOPUS, Web of Science, PubMed, and Google Scholar were searched up to May 13, 2025. Original peer‐reviewed randomized controlled trials (RCTs) in English that investigated patients with periodontitis were included. Study characteristics, methods of ozone application, conclusions, and reported complications were extracted. Pooled mean differences of clinical indices were compared between the ozone therapy groups (adjunctive or monotherapy) and control groups (placebo or CHX). Primary outcomes included clinical indices such as probing pocket depth (PPD), plaque index (PI), and gingival index (GI), whereas secondary outcomes comprised clinical attachment level (CAL), clinical attachment loss (CAtL), bleeding on probing (BoP), and sulcus bleeding index (SBI).

**Results:**

Of the 962 records, 19 clinical studies were included in the qualitative synthesis and 18 RCTs in the quantitative synthesis. For periodontitis, ozonated compounds significantly reduced PPD when used as an adjunct to subgingival instrumentation (MD = −0.780; *p* < 0.0001) and reduced clinical attachment loss (CAL) (MD = −0.457; *p* = 0.001) compared with subgingival instrumentation alone. No statistically significant differences were observed in PI or GI outcomes with ozonated compounds as an adjunct to subgingival instrumentation compared with subgingival instrumentation alone, although sensitivity analyses indicated some instability in the results. Follow‐up periods ranged from 4 to 12 weeks across outcomes (8–12 weeks for GI), with most studies reporting 12‐week follow‐up. No consistent differences were found between ozone and CHX in terms of PPD, CAL, PI or BoP. No adverse effects were reported in the studies.

**Conclusions:**

Ozonated gels and oils were associated with favorable periodontal outcomes, particularly when used as adjuncts to subgingival instrumentation, and no major adverse effects were reported. However, the evidence is limited by short‐term follow‐up and methodological heterogeneity.

## 1 Introduction

Periodontal diseases are chronic inflammatory conditions that affect more than 1 billion people in 2021 (Nascimento et al. 2024). The prevalence of non‐severe periodontitis is approximately 50% among individuals over the age of 65, representing a significant burden on national oral health systems (Periodontal Disease in Adults 2021). The main mechanisms involved in the pathogenesis of periodontitis include bacterial invasion, host immune response, destruction of connective tissue and bone, and an imbalance between tissue damage and repair (Nascimento et al. 2024; Gegout et al. 2023). The immune response, rather than bacterial presence alone, is primarily responsible for the progressive destruction of the supporting structures of the teeth, including the gingiva, periodontal ligament, and alveolar bone (Yekani et al. 2025). Periodontal diseases comprise a spectrum of inflammatory conditions affecting the periodontal tissues and peri‐implant structures. According to contemporary classification frameworks, periodontal health and disease are categorized into distinct entities, including gingivitis, periodontitis—further characterized by staging and grading based on severity, complexity, and risk of progression—and peri‐implant diseases (D′ Ambrosio et al. 2023; Oommen et al. 2024). The gold standard of non‐surgical treatment is subgingival instrumentation, formerly referred to as scaling and root planing (SRP). However, due to complex anatomical areas, residual subgingival bacteria, biofilm resistance, and the need to improve clinical outcomes, various adjunctive antimicrobial agents have been introduced. These include chlorhexidine (CHX) gel (Gegout et al. 2023; Zhao et al. 2020), antibiotics (Gegout et al. 2023), probiotics (Gegout et al. 2023; Mendonça et al. 2024), metformin gel (Kalati et al. 2024), olive oil (Gegout et al. 2023; Mukherjee et al. 2024), and, more recently, different forms of ozonated compounds (Liu et al. 2025).

CHX is included in the EFP guidelines as a short‐term adjunctive antiseptic for plaque control, but it is not the only adjunctive option. The guidelines also conditionally allow selected locally delivered antimicrobials and, in specific cases, systemic antibiotics. Adjunctive modalities such as LASER and antimicrobial photodynamic therapy are mentioned, but due to limited evidence, they are not recommended for routine use. Overall, adjunctive therapies should be applied selectively based on individual clinical indications, highlighting that other agents, including ozone, have not yet been incorporated into standard evidence‐based protocols (Sanz et al. 2020). Despite the increasing clinical use of ozone‐based products in periodontal therapy, there remains no consensus regarding their effectiveness, particularly for ozonated gel and oil formulations, which differ substantially from gaseous or aqueous ozone. Current clinical guidelines do not incorporate ozone‐based therapies, largely due to the absence of a focused synthesis of evidence evaluating these formulations and their comparative efficacy against established adjunctive agents such as chlorhexidine.

Ozone is a triatomic oxygen molecule naturally present in the stratosphere. It rapidly converts to oxygen and exhibits several antibacterial and therapeutic effects in medicine and dentistry. Ozone can damage microbial DNA and cell membranes (Huang et al. 2023), disrupt lipid and protein metabolism (Rangel et al. 2021), and inactivate essential enzymes (Huang et al. 2023). Previous systematic reviews and meta‐analyses on this topic have primarily focused on ozone therapy in the form of gas or water (Gegout et al. 2023; D′ Ambrosio et al. 2023; Oommen et al. 2024; Liu et al. 2025; Moraschini et al. 2020). These studies reported some benefits of ozone therapy, particularly as an adjunctive treatment; however, its efficacy has not been comprehensively compared with established antimicrobials, including CHX.

Ozonated gels and oils provide a longer duration of action against microbes and possess additional healing properties that support soft tissue repair (Russo et al. 2024). Furthermore, these formulations do not produce the adverse effects sometimes associated with gaseous ozone (Veneri et al. 2025). Ozonated gels and oils exhibit antimicrobial, anti‐inflammatory, and regenerative properties (Russo et al. 2024). Local application of these compounds releases reactive oxygen species (ROS) and lipid oxidation products, which exert potent antibacterial, antifungal, and antiviral effects (Rapone et al. 2023). In addition, ozone stimulates local antioxidant enzyme activity, helping to balance oxidative stress in inflamed tissues. Importantly, ozonated formulations promote vasodilation, enhance tissue oxygenation, and stimulate the release of growth factors, such as vascular endothelial growth factor (VEGF) and transforming growth factor‐beta (TGF‐β) (Ucan et al. 2025). These effects improve fibroblast activity, collagen synthesis, and angiogenesis, all of which are critical for soft tissue regeneration and wound healing (Russo et al. 2024; Rapone et al. 2023; Carmo et al. 2025; Patil et al. 2022). Previous systematic reviews and meta‐analyses of clinical trials have generally pooled all forms of ozone therapy, including gaseous ozone, ozonated water, and ozonated gels/oils, and have suggested potential benefits of ozone formulations in periodontal therapy. However, these delivery modalities differ substantially in terms of stability, tissue contact time, application protocols, and clinical practicality. Consequently, combining them within a single analysis may obscure formulation‐specific effects and contribute to the substantial heterogeneity observed across studies. Therefore, the evidence remains inconclusive and does not allow clear conclusions regarding the specific efficacy of ozonated gels and oils (Pardo et al. 2025). In summary, no specific meta‐analysis has yet evaluated ozonated gels and oils, although several recent studies have investigated their efficacy in periodontitis (Mukherjee et al. 2024; Carmo et al. 2025; Patel et al. 2025; Naganandini et al. 2024; Scribante et al. 2024; Dixit et al. 2024; Desingu et al. 2024; Choudhary and Rajasekar 2024; Barahim et al. 2024; Aljaky et al. 2024). Therefore, this study aimed to assess the effectiveness of ozonated gels and oils, both as monotherapy and as an adjunctive treatment, in managing periodontitis.

## 2 Methods

### 2.1 Overview

This study was performed according to Preferred Reporting Items for Systematic Reviews and Meta‐Analyses (PRISMA) guideline and was registered in PROSPERO (code: CRD420251052272) and approved by the Ethics Committee of Mashhad University of Medical Sciences (code: MUMS4040434). In this manuscript, we assess the efficacy of ozonated compounds, including oils and gels, in the management of these conditions, presenting the findings through separate analyses.

### 2.2 Search

The authors (E.P. and M.E.) searched four international databases (PubMed, Embase, SCOPUS, and Web of Science) and the first 200 records of Google Scholar on May 13, 2025. A detailed search strategy is provided in the supporting file (Table S1). Additionally, a hand search of the reference lists and citations of included studies was performed by two authors (N.A. and F.J.) to identify any missing articles.

### 2.3 Selection and Eligibility Criteria

The titles and abstracts were initially screened by two lead authors (E.P. and M.B.). Subsequently, attempts were made to retrieve the full texts of potentially relevant articles, which were then assessed to identify eligible studies. The inclusion criteria were as follows: (population) patients with periodontitis; (intervention) oil or gel formulations of ozonated compounds, administered either as monotherapy or as an adjunct to subgingival instrumentation; (comparison) no intervention, placebo, or CHX; and (outcome) primary outcomes including clinical indices such as PPD, plaque index (PI), and gingival index (GI), with secondary outcomes comprising clinical attachment level (CAL), clinical attachment loss (CAtL), bleeding on probing (BoP), and sulcus bleeding index (SBI). Only original peer‐reviewed studies with randomized controlled or quasi‐experimental designs published in English were considered. Exclusion criteria comprised review articles, animal studies, case reports, and observational studies. Although some eligible clinical trials were identified, only those meeting additional criteria for data completeness and compatibility were included in the meta‐analysis.

### 2.4 Data Extraction

Data were independently extracted by two authors (F.J. and F.A.) and double‐checked by two additional authors (M.E. and M.B.) from the included studies, and are presented in tables. Extracted information included study design, study center, age, sex, sample size, target disease, form of ozone used, methodology (study groups and interventions), study conclusions, clinical indices, and reported complications. Unit‐of‐analysis issues were assessed during data extraction. Only patient‐level outcome data were included in the meta‐analysis.

### 2.5 Quality Assessment

Quality assessment was independently done by three authors (M.E., F.A., and R.A.). The Joanna Briggs Institute (JBI) Critical Appraisal Checklists were used: (1) JBI Critical Appraisal Checklist for randomized controlled trials (RCTs), and (2) JBI Critical Appraisal Checklist for Quasi‐Experimental Studies. Any disagreements at any stage of the study were resolved by discussion between the authors or by consultation with the third author. To enhance the internal validity of the pooled estimates, the meta‐analysis was restricted to studies with moderate to high methodological quality.

### 2.6 Statistical Analysis

The efficacy of ozonated compounds, either as monotherapy or as an adjunct to subgingival instrumentation, was evaluated by comparing clinical indices before and after treatment. In addition, pre‐ and post‐treatment clinical indices were compared between ozonated compounds used as monotherapy or as an adjunct to subgingival instrumentation versus placebo or subgingival instrumentation alone. Moreover, pre‐ and post‐treatment clinical indices were compared between ozonated compounds used as monotherapy or as an adjunct to subgingival instrumentation versus CHX administered as monotherapy or as an adjunct to subgingival instrumentation.

Mean difference (MD) was calculated for uniformly reported outcomes (PPD, BoP, and CAL), while standardized mean difference (SMD) was used for outcomes with varied reporting methods (PI and GI). Effect sizes were calculated based on post‐treatment values, comparing intervention and control groups, rather than changes from baseline, to ensure consistency across studies. Results were presented with 95% confidence intervals in brackets. Subgroup analyses were conducted, when possible, for studies with 12‐week follow‐up, ozone therapy as an adjunctive treatment, and ozone therapy as monotherapy. Sensitivity analyses were performed by switching between random‐ and fixed‐effect models and using the leave‐one‐out method. Heterogeneity was assessed using the I^2^, and the choice between random or fixed‐effect models was based on Cochran's Q test (if the Cochran's Q test P‐value was less than 0.05, a random‐effects model was applied). Publication bias was evaluated using funnel plots, as well as Egger's and Begg's tests. A p‐value of less than 0.05 was considered statistically significant. All analyses were conducted using CMA 3.

## 3 Results

**Figure 1:**
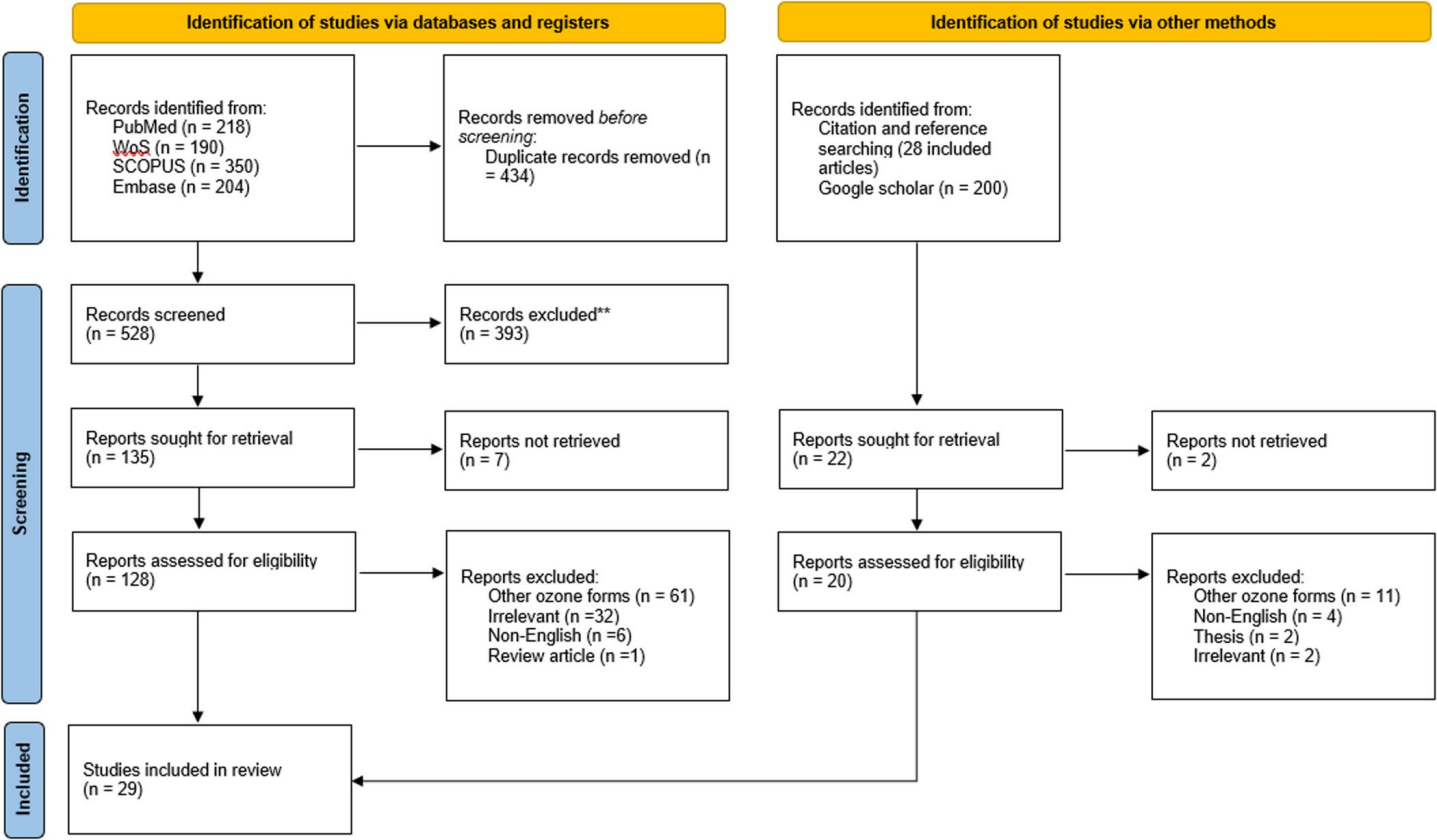
Study selection process illustrated using a PRISMA flowchart.

The initial search yielded a total of 962 records from four international databases, along with 200 additional records identified through the Google Scholar search engine. After full‐text review, 28 studies were deemed eligible for inclusion. An additional study was included through hand searching, bringing the final number of studies included in the qualitative synthesis to 29 (Figure 1). Studies were excluded if they were non‐English (Otero et al. 2020; Menabde et al. 2006), applied different ozone compounds within a single cohort (Rapone et al. 2023), focused solely on antibacterial efficacy (Pietrocola et al. 2018), or assessed peri‐implantitis or gingivitis (Dixit et al. 2024; Choudhary and Rajasekar 2024; Aljaky et al. 2024; AL‐Chalabi and Mohamed 2019; Bulani et al. 2022; Feier et al. 2023; Indurkar and Verma 2016; Butera et al. 2016; Akash et al. 2021; Butera et al. 2023). Finally, 19 studies on periodontitis were included (Mukherjee et al. 2024; Carmo et al. 2025; Patel et al. 2025; Naganandini et al. 2024; Scribante et al. 2024; Desingu et al. 2024; Barahim et al. 2024; Colombo et al. 2021; Gandhi et al. 2019; Rawdy et al. 2020; Lendhey et al. 2020; Nambiar et al. 2022; Patel et al. 2012; Saeed et al. 2017; Tetè et al. 2023; ArvinaRajasekar 2022; Lauritano et al. 2023; Nardi et al. 2020; Shoukheba and Ali 2014). Of these, 17 were RCTs and two were non‐RCTs (Rawdy et al. 2020; Lauritano et al. 2023). One study was excluded from the meta‐analysis due to a duration of less than 3 weeks (Akash et al. 2021), and five studies were excluded due to missing data (Dixit et al. 2024; Desingu et al. 2024; Aljaky et al. 2024; AL‐Chalabi and Mohamed 2019; Lauritano et al. 2023). Ultimately, 19 studies were included in the meta‐analysis. The study characteristics, groups and methods of ozone application, conclusions, and reported complications of the included studies are presented in Table 1 and S3. Table 2 also presents the details of indices by study, groups, and time points. The quality assessment of the included studies is presented in detail in Table S2. Overall, the studies demonstrated moderate to high methodological quality, with clearly defined inclusion criteria, appropriate randomization or quasi‐experimental design, and standardized outcome measurements. Most studies reported adequate follow‐up and low risk of bias in data reporting, indicating that the findings are generally reliable and provide a robust basis for evaluating the effects of ozonated compounds in periodontal therapy.

**Table 1:** Characteristics of included studies.

Author, publication year	Country/center	Study design	Target disease	Ozone form	Method	Sample size (male)	Age (year)
Barahim et al. (2024)	Egypt/Cairo University	RCT	Diabetic patients with stage III periodontitis	Ozonated gel	**Test group:** Subgingival instrumentation + ozone **Control group:** Subgingival instrumentation Ozone gel was administered via a 3 ml disposable plastic syringe	24 (6)	> 18 years old
Carmo et al. (2025)	Brazil/University of Federal Fluminense	RCT	Severe to moderate periodontitis (stage II or III)	Ozonized sunflower seed oil	**Test group:** Subgingival instrumentation + Ozonized oil **Control group:** Subgingival instrumentation + Saline Solution One ml of each substance was administered in the sites after Subgingival instrumentation	16 (7)	57.37 ± 10.05
Colombo et al. (2021)	Italy/University of Pavia	RCT	Periodontitis stage III, grade B	Ozonated gel	**Test group:** Subgingival instrumentation + ozone gel **Control group:** Subgingival instrumentation + CHX gel Gel was applied using syringe	10 (4)	Middle age of 50
Desingu et al. (2024)	India/Vinayaka Mission's Sankarachariyar Dental College	RCT	Chronic periodontitis	Ozonated olive oil	**Test group:** Subgingival instrumentation + Ozonated olive oil **Control group:** Subgingival instrumentation + Gingelly oil After subgingival instrumentation, subgingival ozonated olive oil and gingelly oil were applied. The oil was applied into the periodontal pocket with a blunt disposable tip	10 (4)	25‐45
Sheikh et al. 2020	Egypt/Suez Canal University	Clinical study	Chronic periodontitis	Ozonated olive oil	**Test group:** Subgingival instrumentation + Ozonated olive oil **Control group:** Subgingival instrumentation Ozonated olive oil was administered into the deepest periodontal pocket using a disposable 10 ml syringe. This treatment was applied right after the initial periodontal therapy and repeated on days 7, 14, and 21	24	
Gandhi et al. (2019)	USA/Rutgers School of Dental Medicine	RCT	Moderate to severe chronic periodontitis	Ozonated olive oil	Subgingival instrumentation (ultrasonic and hand instruments in 2 visits) **In second visit:** **Test group:** Subgingival instrumentation + 0.2% subgingival CHX in 2 quadrants **Control group:** Subgingival instrumentation + subgingival ozonated olive oil in other 2 quadrants	25	30‐60
Mukherjee et al. (2024)	India/Rama Dental College Hospital and Research Centre Kanpur	RCT	Chronic periodontitis	Ozonated olive oil	**Group 1:** Subgingival instrumentation + ozonated olive oil **Group 2:** Subgingival instrumentation + plain olive oil **Group 3:** Subgingival instrumentation + CXH gel Subgingival application of the intervention	120 (60)	20‐50
Nambiar et al. (2022)	India/Narayana Dental College and Hospital	RCT	Chronic periodontitis	Ozonated olive oil gel	**Test group:** Subgingival instrumentation + ozonated olive oil **Control group:** Subgingival instrumentation + CHX gel Interventions were introduced into the selected periodontal pockets with blunt cannula until the drugs overflow.	27	38.2
Scribante et al. 2024	Italy/Diagnostic and Pediatric Sciences of the University of Pavia	RCT	Periodontitis (severity I, complexity II)	Ozonated gel	**Test group:** Subgingival instrumentation + ozonated olive oil **Control group:** Subgingival instrumentation + CHX gel Through split‐mouth study, gels were applied to periodontal sites.	30 (15)	59.4 ± 9.32
Tetè et al. (2023)	Italy/Vita‐Salute San Raffaele University	RCT	Periodontal disease	Ozonated gel	**Test group:** Subgingival instrumentation + ozonated olive oil **Control group:** Subgingival instrumentation	30 (12)	—
Lendhey et al. (2020)	India/Dental College and Hospital, Nashik	RCT	Generalized chronic periodontitis	Ozonated oil	**Test group:** Subgingival instrumentation + ozonated olive oil **Control group:** Subgingival instrumentation + ozone oil Oil was applied immediately after the Subgingival instrumentation and after 7, 14 and 21 days.	20	20‐50
Singh et al. 2024	India/NIMS Dental College and Hospital	RCT	Chronic periodontitis	Ozonated oil	**Test group:** Subgingival instrumentation + ozonated oil **Control group:** Subgingival instrumentation + CHX gel Daily application for one month.	56 (42)	45.195
Patel et al. (2012)	India/Kalindi Oro Care & Research Centre	RCT	Chronic periodontitis	Ozonated oil	**Group A:** Subgingival instrumentation **Group B:** Subgingival instrumentation + Ozonated olive oil **Group C:** Ozonated olive oil **Group D:** Topical 1% CHX gluconate gel Sub‐gingival application of treatment oil/gel (treatment was scheduled at baseline, 2, 4, and 6 weeks).	20 (12)	42.64 ± 7.8 (30‐60)
Saeed et al. (2017)	Egypt/Tanta University	RCT	Chronic periodontitis	Ozonated olive oil gel	**Test group:** Subgingival instrumentation + ozonated olive oil gel **Control group:** Subgingival instrumentation + placebo gel	16	> 35
Lauritano et al. (2023)	Italy/University of ferrara	Clinical trial	Chronic periodontitis	Ozonized oral gel	Ozonized oil oral gel Apply the gel once a day after evening oral hygiene	10	35‐55
Shoukheba and Ali (2014)	Egypt/Tanta University	RCT	Localized aggressive periodontitis	Ozonated olive oil gel	**Test group:** Subgingival instrumentation + ozonated olive oil gel **Control group:** Subgingival instrumentation Gel application was performed after initial Subgingival instrumentation and at 1, 2, and 3 weeks	30 (9)	21‐30
Patel et al. (2025)	India/Karnavati University	RCT	Periodontitis Stage II Grade A	Ozonated olive oil	**Test group:** Subgingival instrumentation + ozonated olive oil **Control group:** Subgingival instrumentation + CHX Application of 1 mL of interventions following Subgingival instrumentation.	20	18‐65
Vignesh et al. 2022	India/Saveetha Dental College	RCT	Chronic periodontitis	Ozonated olive oil gel	**Test group:** Subgingival instrumentation + ozonated olive oil gel **Control group:** Subgingival instrumentation + CHX gel After Subgingival instrumentation, a 10 ml sterile plastic syringe with a blunt tip was used to irrigate the periodontal pocket with chlorhexidine and ozonated oil.	20	18‐65
Nardi et al. (2020)	Italy/Sapienza University of Rome	RCT	Chronic periodontitis	Ozonated olive oil	**Test group:** Subgingival instrumentation + ozonated olive oil gel **Control group:** Subgingival instrumentation 12 mL, rinses of 30 s, mouthwash thrice a day for 3 months	96	30‐60

Abbreviations: CHX, chlorhexidine; NR, not reported; RCT, randomized clinical trial.

**Table 2:** Changes in periodontal indices following treatment.

Author, publication year	Group	Time	PPD	CAL	PI	GI	BoP
Barahim et al. (2024)	Subgingival instrumentation + Ozonated gel	Pre	6.44 ± 0.53	7.5 ± 1.78	72.5 ± 15.8^a^,*		61.2 ± 13.4
		3 months	3.11 ± 0.78	4.2 ± 1.87	32.4 ± 9.1		22.6 ± 11.2
		6 months	3.11 ± 0.78	4.11 ± 1.76	19 ± 8.8		9.3 ± 7.6
	Subgingival instrumentation	Pre	6.1 ± 0.32	6.75 ± 1.22	70.2 ± 14.4		58.5 ± 8.7
		3 months	3.8 ± 1.23	4.2 ± 1.38	32 ± 6.2		23.8 ± 7.8
		6 months	3.1 ± 1.1	3.7 ± 1.06	16.2 ± 6.6		10.6 ± 6.8
Carmo et al. (2025)	Subgingival instrumentation + Ozonized oil	Baseline	5.75 ± 1.73	6.06 ± 2.29	56.24 ± 44.25	54.16 ± 36.76	
		3 months	4.75 ± 2.01	5.06 ± 2.29	35.41 ± 46.69	11.45 ± 27.7	
	Subgingival instrumentation + Saline Solution	Baseline	5.2 ± 0.68	5.75 ± 1.87	55.7 ± 40.69	45.83 ± 31.91	
		3 months	4.18 ± 0.83	4.68 ± 1.78	35.41 ± 42.98	17.7 ± 22.33	
Colombo et al. (2021)	Subgingival instrumentation + ozone gel	Baseline	6.21 ± 0.92	6.00 ± 0.83	0.85 ± 0.18^a^	1.67 ± 0.56*	0.43 ± 0.27
		1 month	4.66 ± 0.74	4.42 ± 0.76	0.54 ± 0.09	1.01 ± 0.38	0.15 ± 0.06
		3 months	4.20 ± 0.48	4.32 ± 0.47	0.39 ± 0.07	0.91 ± 0.35	0.09 ± 0.04
	Subgingival instrumentation + CHX gel	Baseline	5.94 ± 0.89	6.13 ± 0.81	0.86 ± 0.16	1.67 ± 0.39	0.33 ± 0.13
		1 month	4.42 ± 0.76	4.85 ± 0.90	0.52 ± 0.07	1.06 ± 0.38	0.11 ± 0.07
		3 months	3.95 ± 0.52	3.99 ± 0.56	0.36 ± 0.08	0.71 ± 0.36	0.09 ± 0.06
Desingu et al. (2024)	Subgingival instrumentation + Ozonated olive oil	Baseline			1.97*	1.8*	
		7 days			1.02	0.9	
	Subgingival instrumentation + Gingelly oil	Baseline			1.66	1.71	
		7 days			0.93	1.06	
Gandhi et al. (2019)	Subgingival instrumentation + Ozonated olive oil	Baseline	5.47 ± 0.32	5.02 ± 0.37 (clinical attachment loss)	1.90 ± 0.12*	1.87 ± 0.12*	
		3 months	3.01 ± 0.40	2.92 ± 0.43	0.66 ± 0.14	0.79 ± 0.12	
	Subgingival instrumentation + CHX	Baseline	5.41 ± 0.32	4.78 ± 0.37	1.86 ± 0.17	1.88 ± 0.13	
		3 months	2.78 ± 0.47	2.63 ± 0.40	0.62 ± 0.21	0.81 ± 0.12	
Mukherjee et al. (2024)	Subgingival instrumentation + subgingival application of ozonated olive oil	Baseline	6.40 ± 0.50	6.00 ± 0.00		2.66 ± 0.38	
		1 month	5.40 ± 0.50	5.00 ± 0.00		0.07 ± 0.05	
	Subgingival instrumentation + subgingival application of plain olive oil	Baseline	6.40 ± 0.50	6.40 ± 0.50		2.65 ± 0.40	
		1 month	5.75 ± 0.44	5.75 ± 0.44		0.35 ± 0.28	
	Subgingival instrumentation + subgingival application of chlorhexidine gel	Baseline	6.30 ± 0.46	6.40 ± 0.50		2.64 ± 0.39	
		1 month	5.28 ± 0.45	5.28 ± 0.45		0.05 ± 0.05	
Nambiar1 et al, 2022	Subgingival instrumentation + ozonated olive oil	Baseline	5.48 ± 0.58	7.78 ± 0.69			1.52 ± 0.50 (sulcus bleeding index)
		3 months	4.19 ± 0.78	6.33 ± 0.92			0.00 ± 0.00
	Subgingival instrumentation + CHX gel	Baseline	5.67 ± 0.55	8.04 ± 0.89			1.52 ± 0.50
		3 months	4.56 ± 0.57	6.89 ± 0.75			0.00 ± 0.000
Tetè et al. (2023)	Subgingival instrumentation + ozonated olive oil	Baseline	5.8		0.56^a^		
		1 month	5		0.37		
		3 months	4		0.22		
		6 months	3.2		0.14		
	Subgingival instrumentation	Baseline	5.4		0.54		
		1 month	5		0.55		
		3 months	4.5		0.34		
		6 months	4.2		0.24		
Lendhey et al. (2020)	Subgingival instrumentation + ozonated olive oil	Baseline	5.2	5.2	2.31*	2.138*	
		1 month	4.2	4.2	1.8	1.319	
	Subgingival instrumentation + ozone oil	Baseline	5.6	5.6	2.02	1.79	
		1 month	5.1	5.1	1.65	1.046	
Singh et al, 2024	Subgingival instrumentation + ozonated oil	Baseline	5.21 ± 0.58	6.32 ± 0.46 (clinical attachment loss)	4.44 ± 0.30^b^	2.22 ± 0.22*	
		1 month	3.04 ± 0.76	4.14 ± 0.68	2.57 ± 0.58	1.18 ± 0.15	
		3 months	3.78 ± 0.36	4.76 ± 0.47	3.31 ± 0.38	1.53 ± 0.27	
	Subgingival instrumentation + CHX gel	Baseline	5.28 ± 0.35	6.38 ± 0.33	4.40 ± 0.2	2.11 ± 0.26	
		1 month	3.10 ± 0.58	4.32 ± 0.57	2.62 ± 0.64	1.18 ± 0.27	
		3 months	4.04 ± 0.56	5.02 ± 0.32	3.31 ± 0.55	1.55 ± 0.28	
Patel et al. (2012)	Subgingival instrumentation	Baseline	8.64 ± 0.386	9.30 ± 0.66	3.36 ± 0.56^b^	2.38 ± 0.36*	4.36 ± 0.27 (sulcus bleeding score 0‐5)
		2 weeks	6.75 ± 0.301	7.05 ± 0.45	2.18 ± 0.72	1.62 ± 0.49	3.27 ± 0.19
		4 weeks	5.04 ± 0.270	5.26 ± 0.74	1.69 ± 0.66	1.12 ± 0.58	2.77 ± 0.16
		6 weeks	4.52 ± 0.488	4.52 ± 0.48	1.29 ± 0.62	1.10 ± 0.37	2.11 ± 0.38
		8 weeks	3.82 ± 0.376	3.87 ± 0.35	0.97 ± 0.71	0.91 ± 0.35	1.67 ± 0.47
	Subgingival instrumentation + ozonated olive oil	Baseline	9.01 ± 0.601	9.02 ± 0.67	3.30 ± 0.59	2.44 ± 0.30	4.43 ± 0.30
		2 weeks	6.83 ± 0.419	6.13 ± 0.98	1.50 ± 0.88	1.10 ± 0.38	2.27 ± 0.46
		4 weeks	3.96 ± 0.400	4.27 ± 0.75	1.01 ± 0.99	0.98 ± 0.57	1.38 ± 0.39
		6 weeks	3.41 ± 0.398	3.60 ± 0.55	0.87 ± 0.70	0.68 ± 0.31	1.09 ± 0.21
		8 weeks	3.06 ± 0.436	3.15 ± 0.43	0.34 ± 0.36	0.27 ± 0.24	0.28 ± 0.22
	Ozonated olive oil	Baseline	8.83 ± 0.542	9.05 ± 0.58	3.22 ± 0.57	2.33 ± 0.40	4.42 ± 0.33
		2 weeks	7.25 ± 0.357	7.30 ± 0.32	2.64 ± 0.45	1.72 ± 0.69	3.52 ± 0.28
		4 weeks	6.01 ± 0.373	6.04 ± 0.36	1.92 ± 0.73	1.35 ± 0.38	2.24 ± 0.29
		6 weeks	4.98 ± 0.311	5.37 ± 0.58	1.69 ± 1.20	0.91 ± 0.37	1.56 ± 0.34
		8 weeks	4.09 ± 0.380	4.21 ± 0.55	1.19 ± 1.16	0.69 ± 0.24	0.87 ± 0.28
	CHX gel	Baseline	8.73 ± 0.731	9.17 ± 0.71	3.32 ± 0.66	2.50 ± 0.33	4.22 ± 0.33
		2 weeks	7.74 ± 0.314	7.85 ± 0.26	2.69 ± 0.55	1.97 ± 0.58	3.84 ± 0.33
		4 weeks	7.02 ± 0.338	7.08 ± 0.29	2.18 ± 0.77	1.51 ± 0.43	2.90 ± 0.31
		6 weeks	5.98 ± 0.418	6.12 ± 0.39	1.51 ± 0.92	1.11 ± 0.43	2.14 ± 0.3
		8 weeks	5.57 ± 0.293	5.68 ± 0.34	1.21 ± 0.87	1.05 ± 0.41	1.51 ± 0.2
Saeed et al. (2017)	Subgingival instrumentation + ozonated olive oil	Baseline	4.5 ± 0.5	2.44 ± 0.5	1.71 ± 0.45		1 ± 0.0
		1 month	2.37 ± 0.9	0.44 ± 0.7	0.65 ± 0.53		0.12 ± 0.35
		3 months	1.5 ± 0.5	0.11 ± 0.33	0.46 ± 0.36		0.25 ± 0.46
		6 months	1.25 ± 0.35	0.11 ± 0.33	0.87 ± 0.46		0.5 ± 0.53
	Subgingival instrumentation	Baseline	4.37 ± 0.51	2.37 ± 0.5	2.09 ± 0.8		1 ± 0.0
		1 month	2.25 ± 0.46	0.25 ± 0.4	1.31 ± 0.70		0.37 ± 0.51
		3 months	2.5 ± 0.7	0.50 ± 0.7	0.39 ± 0.74		0.87 ± 0.35
		6 months	3.25 ± 0.88	0.62 ± 0.5	2.12 ± 0.7		0.87 ± 0.35
Patel et al. (2025)	Subgingival instrumentation + ozonated olive oil	Baseline	5.60 ± 0.69	7.20 ± 0.63 (clinical attachment loss)	1.90 ± 0.31*		1.60 ± 0.51 (sulcus bleeding index)
		3 months	4.50 ± 0.70	6.20 ± 0.63	0.90 ± 0.56		0.60 ± 0.51
	Subgingival instrumentation + CHX	Baseline	5.30 ± 0.48	7.60 ± 0.51	1.80 ± 0.42		1.70 ± 0.48
		3 months	4.30 ± 0.48	6.10 ± 0.87	0.80 ± 0.42		0.30 ± 0.48
Vignesh et al. 2022	Subgingival instrumentation + ozonated olive oil	Baseline	5.50 ± 0.687	5.40 ± 0.655			
		3 weeks	2.20 ± 0.632	3.50 ± 0.655			
	Subgingival instrumentation + CHX	Baseline	5.50 ± 0.687	5.30 ± 0.699			
		3 weeks	2.30 ± 0.699	3.20 ± 0.632			
Nardi et al. (2020)	Subgingival instrumentation + ozonated olive oil	Baseline	4.426 ± 1.391		56.75 ± 25.95		54.67 ± 27.07
		2 weeks	2.351 ± 0.899		18.58 ± 6.503		20.83 ± 8.270
		1 month	2.15 ± 0.922		10.25 ± 8.170		12.67 ± 5.926
		3 months	2.083 ± 0.814		7.417 ± 5.242		7.00 ± 3.377
	Subgingival instrumentation	Baseline	4.394 ± 0.781		77.33 ± 21.10		76.08 ± 27.27
		2 weeks	2.884 ± 0.785		23.75 ± 8.456		27.5 ± 13.31
		1 month	2.206 ± 0.781		14.83 ± 5.684		14.25 ± 7.930
		3 months	2.451 ± 0.746		9.250 ± 3.917		8.25 ± 4.19
Scribante et al. 2024	Ozonated oil	Baseline	6.71 ± 1.03		84.27 ± 16.24		46.14 ± 27.09
		1 month	5.23 ± 1.05		45.63 ± 17.05		21.73 ± 13.66
		3 months	4.33 ± 1.13		45.10 ± 18.23		14.08 ± 9.15
		6 months	3.89 ± 1.17		42.03 ± 21.13		10.78 ± 7.72
		9 months	3.40 ± 0.80		36.07 ± 19.41		7.60 ± 6.61
		12 months	3.34 ± 0.77		32.70 ± 16.86		5.80 ± 5.49
		15 months	3.29 ± 0.78		29.50 ± 16.21		4.95 ± 5.49
		18 months	3.15 ± 0.75		25.93 ± 15.35		4.40 ± 4.87
		21 months	3.05 ± 0.67		23.73 ± 14.86		3.57 ± 4.25
		24 months	3.01 ± 0.64		21.20 ± 14.02		3.33 ± 4.01
	CHX	Baseline	6.49 ± 0.91		84.90 ± 16.70		44.60 ± 26.90
		1 month	5.15 ± 0.89		49.90 ± 1.80		27.10 ± 17.20
		3 months	4.57 ± 1.01		50.10 ± 16.50		18.40 ± 12.30
		6 months	4.30 ± 0.94		47.20 ± 19.40		16.10 ± 10.20
		9 months	4.03 ± 0.75		42.90 ± 18.30		14.10 ± 9.00
		12 months	4.00 ± 0.71		41.40 ± 17.40		13.90 ± 8.73
		15 months	3.92 ± 0.73		40.10 ± 16.60		13.80 ± 9.05
		18 months	3.85 ± 0.72		40.30 ± 15.30		13.40 ± 8.76
		21 months	3.79 ± 0.73		38.40 ± 13.90		13.20 ± 8.38
		24 months	3.77 ± 0.73		37.30 ± 14.40		13.30 ± 8.46

Abbreviations: BI, bleeding index; CAL, clinical attachment level; CAtL, clinical attachment loss; GI, gingival index; PI, plaque index; PPD, periodontal pocket depth; SI, suppuration index; TM, tooth mobilit.

PI measurement methods: *Silness & Löe, *Löe & Silness, ⁑Full Mouth Plaque Score; GI measurement methods.

^a^ O'Leary's,

^b^ Turesky (Quigley–Hein),

### 3.1 PPD

**Figure 2:**
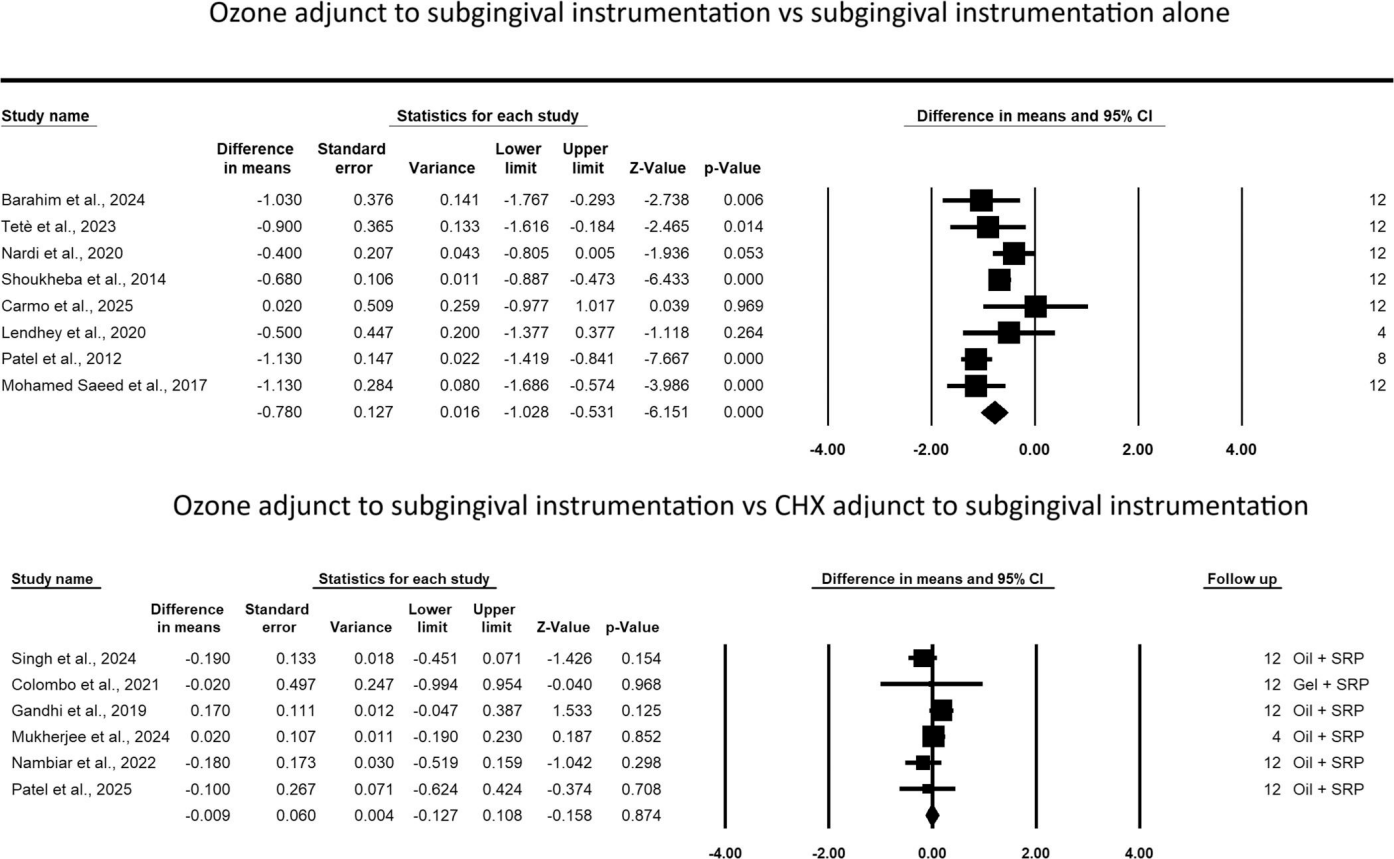
Comparison of probing pocket depth (PPD) between subgingival instrumentation alone and subgingival instrumentation with adjunctive ozonated compounds.

A total of 18 studies reported outcomes on PPD following treatment with ozonated compounds, including 339 patients in the ozone therapy groups and 339 in the control groups. Compared with subgingival instrumentation alone, ozonated compounds as an adjunct to subgingival instrumentation significantly reduced PPD (MD = −0.780 mm; 95% CI: −1.028 to −0.531; *p* < 0.0001; I^2^ = 52%) (Figure 2). There was no significant difference between ozonated compounds and CHX as adjuncts to subgingival instrumentation in terms of PPD reduction (MD = –0.009 mm; 95% CI: –0.127 to 0.108; *p* = 0.874; I^2^ = 11%) (Figure 2). Subgroup analyses for all indices are presented in Table 3. All analyses, including meta‐regression, subgroup and sensitivity analyses, are included in the supporting file.

**Table 3:** Results of the meta‐analysis for periodontitis indices.

Index	Test	Comparison	Mean	Lower limit	Upper limit	*p*Value	I2 (%)	Analysis model
PPD	Adjunctive ozone therapy	—	−2.130	−2.882	−1.377	< 0.0001	99	R
	Ozone monotherapy	—	−3.119	−6.343	0.105	0.058^a^	98	R
	Subgroup (12 weeks follow‐up)	—	−1.882	−2.402	−1.362	< 0.0001	97	R
	Adjunctive ozone therapy	Subgingival instrumentation	−0.780	−1.028	−0.531	< 0.0001	53	R
	Subgroup (12 weeks follow‐up)	Subgingival instrumentation	−0.683	−0.847	−0.520	< 0.0001	33	F
	Adjunctive ozone therapy	Adjunctive CHX	−0.009	−0.127	0.108	0.874	11	F
	Ozone monotherapy	CHX monotherapy	−0.986	−2.333	0.362	0.152	82	R
	Subgroup (12 weeks follow‐up)	CHX	−0.026	−0.167	0.115	0.719	10	F
PI	Adjunctive ozone therapy	—	−3.192	−4.363	−2.021	< 0.0001	93	R
	Ozone monotherapy	—	−1.591	−2.564	−0.618	0.001	88	R
	Adjunctive ozone therapy	Subgingival instrumentation	−0.222	−0.535	0.091	0.165^b^	43	F
	Subgroup (12 weeks follow‐up)	Subgingival instrumentation	0.028	−0.362	0.157	0.292	0	F
	Adjunctive ozone therapy	Adjunctive CHX	−0.013	−0.321	0.295	0.932	49	F
	Ozone monotherapy	CHX monotherapy	−0.213	−0.593	0.166	0.271	62	R
	Subgroup (12 weeks follow‐up)	CHX	−0.182	−0.520	0.157	0.292	0	F
GI	Adjunctive ozone therapy	—	−3.282	−4.688	−1.876	< 0.0001	91	R
	Ozone monotherapy	—	−2.281	−3.181	−1.380	< 0.0001	78	R
	Adjunctive ozone therapy	Subgingival instrumentation	−0.922	−2.180	0.336	0.151	80	R
	Adjunctive ozone therapy	Adjunctive CHX	−0.163	−0.479	0.152	0.311	0	F
CAL	Adjunctive ozone therapy	—	−2.469	−3.850	−1.088	< 0.0001	97	R
	Ozone monotherapy	—	−3.145	−6.535	0.245	0.069	97^a^	R
	Subgroup (12 weeks follow‐up)	—	−2.110	−2.739	−1.481	< 0.0001	79	R
	Adjunctive ozone therapy	Subgingival instrumentation	−0.457	−0.727	−0.187	0.001	0	F
	Subgroup (12 weeks follow‐up)	Subgingival instrumentation	−0.473	−0.935	−0.01	0.045	0	F
	Adjunctive ozone therapy	Adjunctive CHX	0.277	−0.209	0.763	0.264	0	F
	Ozone monotherapy	CHX monotherapy	−1.282	−1.636	−0.928	< 0.0001	51	F
BoP	Adjunctive ozone therapy	—	−1.881	−2.268	−1.494	< 0.0001	25	F
	Adjunctive ozone therapy	Subgingival instrumentation	−0.286	−1.590	1.018	0.667^b^	89	R
SBI	Adjunctive ozone therapy	Adjunctive CHX	−5.241	−8.420	−2.062	0.001	93	R

Sensitivity analysis:

^a^ Fixed‐effect analysis showed different results.

^b^ Leave‐one out analysis showed different results.

### 3.2 Pi

**Figure 3:**
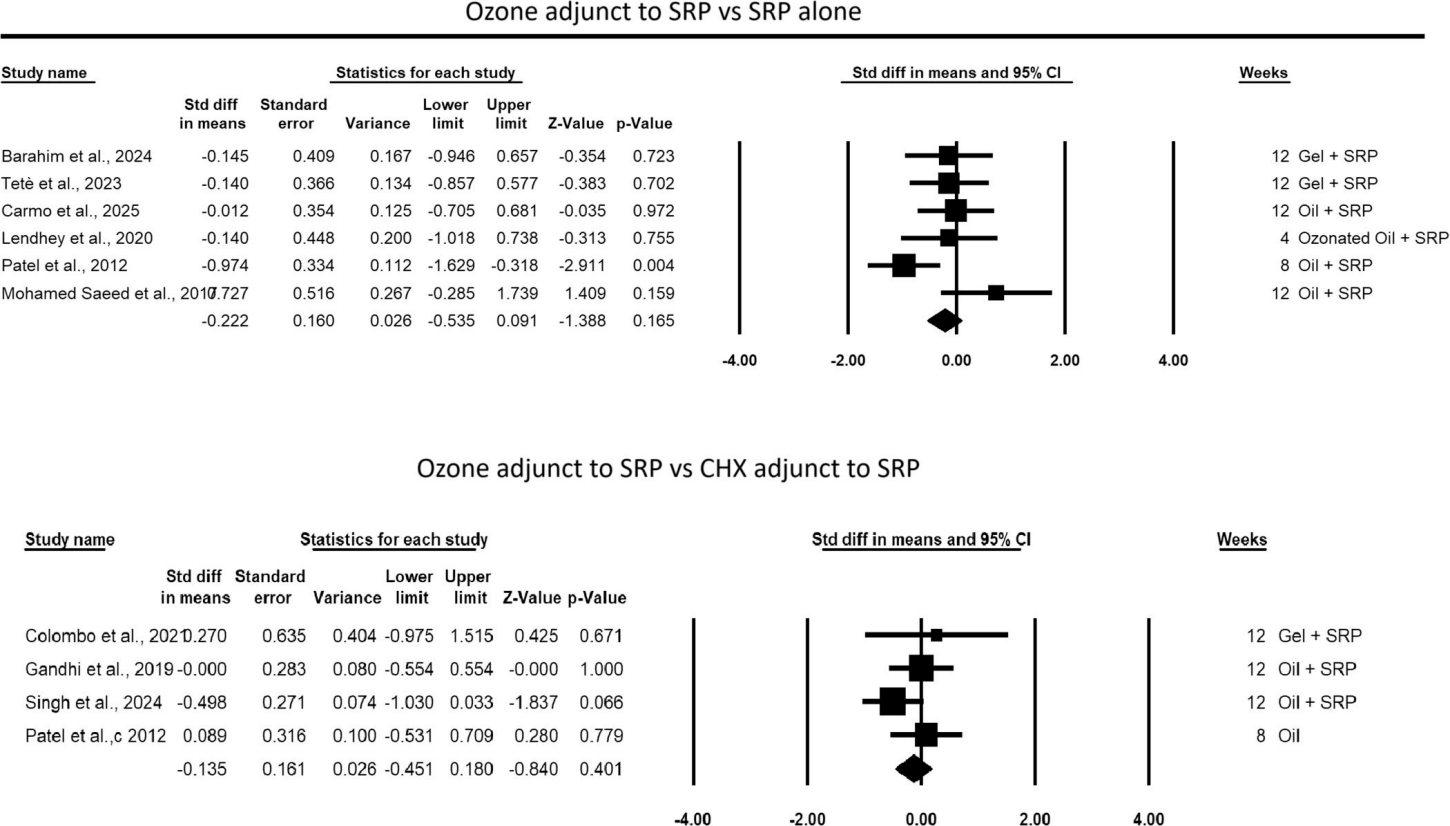
Comparison of plaque index (PI) outcomes between subgingival instrumentation alone and subgingival instrumentation with adjunctive ozonated compounds.

Seventeen studies provided data on PI after treatment with ozonated compounds, encompassing 289 participants in each of ozone therapy groups and control groups. Subgingival instrumentation alone and ozonated compounds as an adjunct to subgingival instrumentation reached similar results in PI outcomes (SMD = −0.222; 95% CI: −0.535 to 0.091; *p* = 0.165; I^2^ = 41%) (Figure 3). Sensitivity analyses, including the use of a random‐effects model and leave‐one‐out method, confirmed the stability of the results. Moreover, no significant difference was observed between CHX as an adjunct to subgingival instrumentation and ozonated compounds as an adjunct to subgingival instrumentation in terms of PI changes following treatment (SMD = –0.135; 95% CI: –0.451 to 0.180; *p* = 0.401; I^2^ = 0%) (Figure 3). Sensitivity analyses showed the robustness of these results.

### 3.3 GI

**Figure 4:**
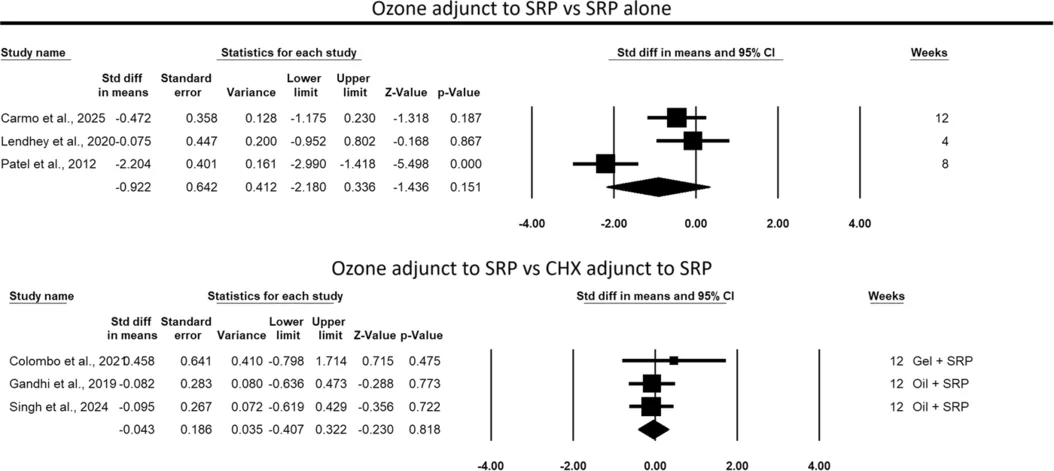
Comparison of gingival index (GI) outcomes between subgingival instrumentation alone and subgingival instrumentation with adjunctive ozonated compounds.

Twelve studies provided data on GI after treatment with ozonated compounds, each involving 229 participants in the ozone therapy and control groups. Ozone therapy as an adjunct to subgingival instrumentation, in the form of gel or oil, was associated with a non‐significant standardized reduction in GI compared with subgingival instrumentation alone (SMD = −0.922; 95% CI: −2.180 to 0.336; *p* = 0.151; I^2^ = 80%) (Figure 4). Additionally, ozonated compounds as an adjunct to subgingival instrumentation showed no significant difference in GI outcomes compared with CHX as an adjunct to subgingival instrumentation (SMD = −0.043; 95% CI: −0.407 to 0.322; *p* = 0.818; I^2^ = 0%) (Figure 4).

### 3.4 CAL

A total of nine studies reported outcomes on CAL following treatment with ozonated compounds, including 116 patients in each of the ozone therapy groups and control groups. Ozonated compounds as an adjunct to subgingival instrumentation were more effective than subgingival instrumentation alone in reducing CAL (MD = −0.457 mm; 95% CI: −0.727 to −0.187; *p* = 0.001; I^2^ = 0%). No significant difference was observed in CAL changes following treatment with ozonated compounds as an adjunct to subgingival instrumentation compared with CHX as an adjunct to subgingival instrumentation (MD = 0.277 mm; 95% CI: −0.209 to 0.763; *p* = 0.264; I^2^ = 0%).

### 3.5 BoP and SBI

Six and four studies evaluated BoP and SBI after treatment with ozonated compounds, involving 103 and 77 patients in each of the ozone therapy and control groups, respectively. Both random‐ and fixed‐effects models showed no statistically significant difference in BoP reduction. The point estimate from the random‐effects model slightly favored the ozone adjunct group (SMD = –0.286; 95% CI: –1.590 to 1.018; *p* = 0.667; I^2^ = 89%), while the fixed‐effects model estimate slightly favored the control (fixed‐effects: SMD = 0.343; 95% CI: –0.008 to 0.693; *p* = 0.055). This inconsistency is likely due to high heterogeneity (I^2^ = 89%) and the influence of individual studies, as indicated by the leave‐one‐out analysis. Removal of the study by Nardi et al. resulted in a significant difference favoring ozonated compounds combined with subgingival instrumentation over subgingival instrumentation alone (SMD = −0.765; 95% CI: −1.419 to −0.112; *p* = 0.022; I^2^ = 62%) (Nardi et al. 2020).

Overall, in patients with periodontitis, ozonated compounds used as an adjunct to subgingival instrumentation were associated with significant improvements in PPD and CAL compared with subgingival instrumentation alone. In contrast, no significant differences were observed for PI, GI, or BoP/SBI. Furthermore, ozonated compounds demonstrated efficacy comparable to that of CHX when used as an adjunct to subgingival instrumentation across the evaluated periodontal indices. Key findings and reported complications of the included studies are presented in Table S3.

## 4 Discussion

This study is a systematic review and meta‐analysis of randomized and quasi‐experimental clinical trials evaluating the efficacy of ozonated gels and oils in the treatment of periodontitis, providing formulation‐specific evidence for these ozone delivery modalities. Our findings suggest that ozonated compounds may be more effective as an adjunct to subgingival instrumentation, resulting in greater short‐term reductions in PPD and CAL and more favorable GI outcomes compared with subgingival instrumentation alone. In addition, ozonated compounds appeared to demonstrate comparable short‐term effectiveness to CHX, with similar effects on PPD, PI, and GI outcomes, while potentially offering greater benefit than CHX monotherapy for CAL and than adjunctive CHX therapy for SBI. However, these observations should be interpreted with caution given the variability among studies and the limitations of the available evidence. No studies reported complications related to ozone therapy.

A meta‐analysis of RCTs by Sareen et al. showed the impact of ozone therapy, in the form of gas and water, as an adjunctive to subgingival instrumentation on improving periodontal indices. They reported significantly better PPD and GI outcomes compared with subgingival instrumentation alone (Sareen et al. 2025). In a recent meta‐analysis by Liu et al. in 2025, three forms of ozone therapy (gas, water and gel) were evaluated for the treatment of periodontitis (Liu et al. 2025). In agreement with our findings, they reported that ozone therapy as an adjunctive treatment was associated with improved PPD and GI outcomes, with no significant difference observed for PI. However, our analysis also showed that CAL and BoP were significantly reduced with ozone therapy as an adjunctive treatment compared with subgingival instrumentation alone. Moreover, a 2020 meta‐analysis concluded that ozone therapy is not a suitable adjunctive treatment to subgingival instrumentation for managing periodontitis (Moraschini et al. 2020). Our analysis focused specifically on ozonated gels and oils, whereas the 2020 meta‐analysis included studies using gaseous or aqueous ozone, which may have different pharmacokinetic and clinical properties. Differences in delivery methods (topical gel application *vs.* gaseous or aqueous irrigation) may influence tissue contact time, penetration into periodontal pockets, and overall therapeutic effect. Additionally, our study conducted a more comprehensive and updated search, resulting in a higher number of included studies (23 *vs.* 10). Moreover, follow‐up duration varied considerably among studies, ranging from short‐term assessments to longer observation periods, which may particularly affect outcomes such as CAL and BoP that require sustained healing to demonstrate measurable improvement. To the best of our knowledge, no clinical study has directly compared these different ozone forms for periodontal treatment. Our findings, together with theoretical considerations, suggest that oil‐based gel formulations may offer greater stability and longer surface contact time, which could contribute to improved CAL and BoP outcomes. However, these hypotheses remain speculative and require confirmation through direct comparative clinical trials (Russo et al. 2024). In vitro and preclinical studies suggest that ozonated oils may maintain antimicrobial activity for longer periods and promote fibroblast proliferation and collagen synthesis. These mechanistic observations provide biological plausibility but do not constitute direct clinical evidence of periodontal repair (Ugazio et al. 2020; Kim et al. 2009). The hydrophobic nature of oil‐based formulations also allows them to penetrate and adhere to gingival and subgingival areas more effectively than water and gaseous forms. Future studies are needed to confirm these observations and determine the most effective ozone form for periodontitis treatment. Moreover, recent meta‐analyses showed the efficacy of CHX in improving PPD, GI, and CAL when used as an adjunctive therapy (Gegout et al. 2023; Zhao et al. 2020).

According to our findings, ozonated compounds demonstrated comparable effects to CHX for certain clinical indices. In some outcomes, point estimates favored ozonated compounds; however, these findings should be interpreted cautiously due to overlapping confidence intervals and variability among studies.

Today, subgingival instrumentation is considered the gold standard of periodontitis treatment due to its effectiveness in improving clinical indices such as PI, BOP, CAL and PPD. It has been proven that, in addition to systemic factors, local factors related to the 3D configuration of the periodontal lesion also influence the results of subgingival instrumentation (Suvan et al. 2020). Therefore, different gel preparations have been investigated to improve the removal of subgingival biofilm and support the restoration of tissue homeostasis. Among the various gels investigated for adjunctive therapy, some antibiotics showed significant efficacy, and the use of CHX is suggested as an adjunctive aid for periodontitis management (Gegout et al. 2023; Sanz et al. 2020). Although statistically significant improvements were observed for certain outcomes, their clinical relevance should be interpreted cautiously. For example, the pooled reduction in PPD associated with adjunctive ozonated compounds was less than 1 mm, and the observed improvement in CAL was also modest. While such differences may be beneficial in specific clinical situations, their practical significance in routine periodontal therapy remains uncertain. Clinicians should consider not only statistical significance but also factors such as treatment cost, product availability, patient compliance, ease of application, and the magnitude of benefit relative to established adjunctive therapies. Importantly, ozonated compounds did not demonstrate clear superiority over CHX in most evaluated outcomes. Therefore, although ozone‐based formulations appear promising, current evidence does not support their routine replacement of established adjunctive therapies, and treatment decisions should be individualized according to patient and clinical needs. According to the current European Federation of Periodontology (EFP) guidelines for the treatment of stages I–III periodontitis, ozone therapy is not included among the recommended treatment modalities. These guidelines serve as the primary reference for clinicians in selecting evidence‐based strategies. The absence of ozone and its derivatives in these recommendations emphasizes that, although our findings suggest promising adjunctive benefits, further robust and long‐term RCTs are required before ozone‐containing compounds can be considered for inclusion in international treatment protocols (Sanz et al. 2020). According to our findings, ozonated compounds may provide favorable effects on PI and GI outcomes when used either as monotherapy or as an adjunct to subgingival instrumentation. However, adjunctive therapy demonstrated substantially larger effect sizes than monotherapy, and monotherapy did not significantly improve deeper periodontal parameters such as PPD and CAL (PI: adjunctive, SMD = −3.192 [95% CI: −4.363, −2.021]; monotherapy, SMD = −1.591 [95% CI: −2.564, −0.618]; GI: adjunctive, SMD = −3.282 [95% CI: −4.688, −1.876]; monotherapy, SMD = −2.281 [95% CI: −3.181, −1.380]). This may be attributed to the fact that subgingival instrumentation primarily disrupts biofilms mechanically, while ozonated compounds provide additional antimicrobial, anti‐inflammatory, and healing‐promoting effects (Liu et al. 2025). These findings reinforce the role of ozonated gel or oil as a supportive therapy rather than a standalone treatment, particularly in cases of moderate to severe periodontitis where mechanical biofilm disruption alone may be insufficient to achieve optimal healing.

CHX is widely used in dentistry as an adjunctive antiseptic, available in various formulations such as mouth rinses, gels, and slow‐release chips, and in different concentrations. Its use has been associated with adverse effects, including tooth discoloration, altered taste sensation, and mucosal irritation (Poppolo Deus and Ouanounou 2022). Ozone is applied in periodontal therapy in multiple forms, including gaseous ozone, ozonated gels, and ozonated oils. While gaseous ozone carries potential toxicity risks if inhaled (Veneri et al. 2025), the included studies investigating ozonated gel and oil formulations reported good tolerability, with no adverse effects observed. Application was generally local, either as a topical gel/oil within the periodontal pocket or as an adjunct to subgingival instrumentation. However, the limited sample sizes and short follow‐up periods should be considered when interpreting safety data. Other adjunctive therapies used in periodontal treatment, such as locally delivered antibiotics, slow‐release antiseptic chips, and antimicrobial photodynamic therapy, have shown variable efficacy and may be associated with undesirable effects depending on the formulation and method of application (Gegout et al. 2023). Clearly specifying the form and mode of administration is critical for interpreting clinical outcomes.

The observed heterogeneity indicates that treatment effects may vary across different clinical settings and patient populations. Therefore, the pooled estimates should be interpreted as average effects rather than precise estimates applicable to all treatment protocols, particularly for outcomes such as GI and BoP that demonstrated inconsistency across analyses.

A comprehensive literature search with subgroup and sensitivity analyses is a major strength of this study. However, several limitations should be noted, each of which may affect the internal validity and external generalizability of the conclusions. First, the relatively small sample sizes in many included studies reduce statistical power and increase the risk of imprecise effect estimates. Second, the short follow‐up duration of several trials restricts the ability to draw firm conclusions regarding the long‐term efficacy and safety of ozonated therapies. Third, the limited number of eligible studies for certain subgroup analyses precludes definitive conclusions and increases the likelihood that observed effects may be driven by individual studies rather than consistent evidence. Fourth, the substantial heterogeneity observed in some analyses, likely stemming from variations in study design, sample characteristics, ozone formulations, and intervention protocols, further reduces the certainty and transferability of pooled estimates. Additionally, the lack of standardized definitions and assessment protocols for clinical outcomes across included studies limits direct comparability between trials and likely contributes to the observed heterogeneity, thereby reducing the reliability and interpretability of pooled effect estimates. Fifth, although the use of standardized mean differences enabled quantitative synthesis across studies using different measurement scales, it limits the direct clinical interpretability of outcomes—particularly for PI and GI. Sixth, the inclusion of studies assessing ozonated compounds as monotherapy reflects available evidence but may have limited direct clinical applicability in periodontal therapy, where adjunctive use is more commonly practiced. Finally, despite conducting subgroup and sensitivity analyses to test robustness, some outcomes (notably GI and BoP) demonstrated inconsistency depending on the statistical model or the influence of individual studies. Consequently, these findings should be interpreted with caution.

Future research should focus on conducting large, high‐quality RCTs with standardized ozone protocols for comparing different forms of ozone. Further studies should also investigate the biological mechanisms of action, assess long‐term safety, and compare ozone‐containing compounds with other adjunctive therapies to better define their clinical value. Additionally, future meta‐analyses would benefit from individual patient data (IPD) to better account for heterogeneity and identify patient subgroups that may respond more favorably to ozone therapy.

## 5 Conclusions

This systematic review and meta‐analysis indicates that ozonated gels and oils may offer potential clinical benefits in the management of periodontitis. When used as an adjunct to subgingival instrumentation, ozonated formulations were associated with improvements in clinical parameters, including PPD, CAL, GI, and BoP, primarily assessed at short‐term follow‐up. In contrast, evidence regarding their use as monotherapy remains limited and inconclusive, and does not support clinical recommendation. In several studies, ozonated compounds showed effects comparable to CHX when used as adjunctive agents, and no significant adverse events were reported. Nevertheless, these findings should be interpreted with caution due to methodological limitations, including heterogeneity in study design, variability in ozone formulations and application protocols, limited sample sizes, and short follow‐up periods. At present, ozonated gels and oils should be regarded as promising adjunctive therapeutic options rather than established alternatives to CHX. Further large‐scale, well‐designed RCTs with standardized outcome measures and long‐term follow‐up are required to confirm their efficacy, safety, and comparative effectiveness.

## Author Contributions

E.P., M.E., and M.B. developed the study concept and carried out the literature search. M.B. and F.M. screened and selected the eligible studies. F.J. and F.A. collected the data. S.H., N.A., and A.A. conducted the statistical analyses. E.P., M.E., and M.B. assessed and interpreted the findings. All authors participated in drafting and revising the manuscript.

## Funding

The authors have nothing to report.

## Ethics Statement

This study was approved by the Research Ethics Committee of Mashhad University of Medical Sciences (IR.MUMS.DENTISTRY.REC.1404.074). All authors have read and approved the final manuscript and consent to its publication.

## Conflicts of Interest

The authors declare no conflicts of interest.

## Supporting information


Supporting File 1

## Data Availability

The data that support the findings of this study are available in the supporting material of this article. The datasets generated and/or analyzed during the current study are available from the corresponding author on reasonable request.
