# Supplementary material for: Efficacy of Ozonated Gels and Oils As Adjunctive or Monotherapy in Periodontitis: A Systematic Review and Meta‐Analysis of Randomized Controlled Trials
Source: Clin Exp Dent Res. 2026 Aug 25;12(4):e70439. doi: 10.1002/cre2.70439 (PMC13506133; doi:10.1002/cre2.70439)
Supplement: Supplementary file 1 [file CRE2-12-e70439-s001.docx]

|  | **Table S1: Search strategy** | |
| --- | --- | --- |
|  | **PubMed (12 May 2025)** | |
| **results** | **Query** | **Search** |
| 180,739 | ("Periodontal Diseases"[Mesh] OR “Parodont*”[Title/Abstract] OR "Periodont*"[Title/Abstract] OR "Pyorrhea Alveolaris"[Title/Abstract] or gingiv*[Title/Abstract] or "DMF Index"[Title/Abstract] or "Oral Hygiene Index"[Title/Abstract] OR "Cavitas Oris"[Title/Abstract] OR "Cavitas oris propria"[Title/Abstract] OR "Mouth Cavity Proper"[Title/Abstract] OR “peri implant*”[Title/Abstract] OR “peri-implant*”[Title/Abstract] OR “Plaque Index”[Title/Abstract] OR “Gingival Index” [Title/Abstract] OR “Clinical Attachment Level” [Title/Abstract] OR “Pocket Depth” [Title/Abstract] OR “clinical attachment loss”[Title/Abstract]) | **#1** (Periodontal disease) |
| 64,123 | (Ozone[Mesh] or Ozon*[Title/Abstract] OR "greenhouse gas*"[Title/Abstract] OR “o3”[title/abstract] OR “triatomic oxygen”[title/abstract] OR “Bio-ozon*”[Title/Abstract] OR “Bioozon*”[Title/Abstract] OR “GeliO3*”[Title/Abstract] OR Ozoral[Title/Abstract]) | **#2** (Ozone) |
| **218** | **#1 AND #2** | **Final** |
|  | **Scopus (12 May 2025)** | |
| 235,458 | TITLE-ABS-KEY("Periodontal Diseases" OR “Parodont*” OR "Periodont*" OR "Pyorrhea Alveolaris" or gingiv* or "DMF Index" or "Oral Hygiene Index" OR "Cavitas Oris" OR "Cavitas oris propria" OR "Mouth Cavity Proper" OR “peri implant*” OR “peri-implant*” OR “Plaque Index” OR “Gingival Index” OR “Clinical Attachment Level” OR “Pocket Depth” OR “clinical attachment loss”) | **#1** (Periodontal disease) |
| 360,731 | TITLE-ABS-KEY(“Ozone” OR “Ozon*” OR "greenhouse gas*" or “o3” OR “triatomic oxygen” OR “Bio-ozon*” OR “Bioozon*” OR “GeliO3*” OR “Ozoral”) | **#2** (Ozone) |
| **350** | **#1 AND #2** | **Final** |
|  | **Embase (13 May 2025)** | |
| 158,434 | ("Periodontal Diseases":ti,ab OR “Parodont*”:ti,ab OR "Periodont*":ti,ab OR "Pyorrhea Alveolaris":ti,ab or “gingiv*”:ti,ab or "DMF Index":ti,ab or "Oral Hygiene Index":ti,ab OR "Cavitas Oris":ti,ab OR "Cavitas oris propria":ti,ab OR "Mouth Cavity Proper":ti,ab OR “peri implant*”:ti,ab OR “peri-implant*”:ti,ab OR “Plaque Index”:ti,ab OR “Gingival Index”:ti,ab OR “Clinical Attachment Level”:ti,ab OR “Pocket Depth”:ti,ab OR “clinical attachment loss”:ti,ab) | **#1** (Periodontal disease) |
| 68,042 | (Ozone:ti,ab or Ozon*:ti,ab OR "greenhouse gas*":ti,ab OR “o3”:ti,ab OR “triatomic oxygen”:ti,ab OR “Bio-ozon*”:ti,ab OR “Bioozon*”:ti,ab OR “GeliO3*”:ti,ab OR Ozoral:ti,ab) | **#2** (Ozone) |
| **204** | **#1 AND #2** | **Final** |
|  | **Web Of Science (13 May 2025)** | |
| 132,458 | ((TI=("Periodontal Diseases" OR “Parodont*” OR "Periodont*" OR "Pyorrhea Alveolaris" or gingiv* or "DMF Index" or "Oral Hygiene Index" OR "Cavitas Oris" OR "Cavitas oris propria" OR "Mouth Cavity Proper" OR “peri implant*” OR “peri-implant*” OR “Plaque Index” OR “Gingival Index” OR “Clinical Attachment Level” OR “Pocket Depth” OR “clinical attachment loss”)) OR (AB=("Periodontal Diseases" OR “Parodont*” OR "Periodont*" OR "Pyorrhea Alveolaris" or gingiv* or "DMF Index" or "Oral Hygiene Index" OR "Cavitas Oris" OR "Cavitas oris propria" OR "Mouth Cavity Proper" OR “peri implant*” OR “peri-implant*” OR “Plaque Index” OR “Gingival Index” OR “Clinical Attachment Level” OR “Pocket Depth” OR “clinical attachment loss”))) | **#1** (Periodontal disease) |
| 234,282 | ((TI=(Ozone OR Ozon* OR "greenhouse gas*" or “o3” OR “triatomic oxygen” OR “Bio-ozon*” OR “Bioozon*” OR “GeliO3*” OR Ozoral)) OR (AB=( Ozone OR Ozon* OR "greenhouse gas*" or “o3” OR “triatomic oxygen” OR “Bio-ozon*” OR “Bioozon*” OR “GeliO3*” OR Ozoral))) | **#2** (Ozone) |
| **190** | **#1 AND #2** | **Final** |

Table S2. Quality assessment of the included studies using the JBI checklist (A: RCTs, B: Quasi experimental studies)

A:

| **A: Randomized clinical study** | | | | | | | | | | | | | |
| --- | --- | --- | --- | --- | --- | --- | --- | --- | --- | --- | --- | --- | --- |
| **Study** | **Was true randomization used for assignment of participants to treatment groups?** | **Was allocation to treatment groups concealed?** | **Were treatment groups similar at the baseline?** | **Were participants blind to treatment assignment?** | **Were those delivering treatment blind to treatment assignment?** | **Were outcomes assessors blind to treatment assignment?** | **Were treatment groups treated identically other than the intervention of interest?** | **Was follow up complete and if not, were differences between groups in terms of their follow up adequately described and analyzed?** | **Were participants analyzed in the groups to which they were randomized?** | **Were outcomes measured in the same way for treatment groups?** | **Were outcomes measured in a reliable way?** | **Was appropriate statistical analysis used?** | **Was the trial design appropriate, and any deviations from the standard RCT design (individual randomization, parallel groups) accounted for in the conduct and analysis of the trial?** |
| **Barahim et al., 2024** | Y | Y | Y | N | N | Y | Y | N | Y | Y | Y | Y | Y |
| **Bulani et al., 2022** | Y | Y | Y | Y | Y | Y | Y | Y | Y | Y | Y | Y | Y |
| **Butera et al., 2023** | Y | Y | Y | Y | N | N | Y | Y | Y | Y | Y | Y | Y |
| **Carmo et al., 2025** | Y | U (N) | Y | N | N | Y | Y | Y | Y | Y | Y | Y | Y |
| **Choudhary & Rajasekar, 2024** | Y | Y (U) | Y | Y | Y | Y | Y | U | Y | Y | Y | Y | Y |
| **Colombo et al., 2021** | Y | Y | Y | Y | Y | Y | Y | Y | Y | Y | Y | Y | Y |
| **Desingu et al., 2024** | U | U | Y | U | U | U | Y | U | U | Y | U | Y | U |
| **Dixit et al., 2024** | Y | U | Y | U | U | U | Y | U | U | Y | Y | Y | Y |
| **Sheikh et al., 2020** | Y | U | Y | N | N | N | Y | Y | Y | Y | Y | Y | Y |
| **Feier et al., 2024** | Y | Y | Y | Y | Y | Y | Y | U | U | Y | U | Y | Y |
| **Gandhi et al., 2019** | Y | U | N | U | U | U | Y | Y | Y | Y | Y | Y | Y |
| **Indurkar et al., 2016** | Y | N | Y | N | N | N | Y | U | Y | Y | U | Y | Y |
| **Mukherjee et al., 2024** | Y | U | Y | U | U | U | Y | Y | Y | Y | Y | Y | Y |
| **Nambiar et al., 2022** | Y | U | Y | Y | Y | Y | Y | N | Y | Y | Y | Y | Y |
| **Scribante et al., 2023** | Y | Y | Y | N | N | Y | Y | Y | Y | Y | Y | Y | Y |
| **Tetè et al., 2023** | Y | U | Y | N | N | Y | Y | Y | Y | Y | Y | Y | Y |
| **Lendhey et al., 2020** | Y | U | Y | U | U | U | Y | Y | Y | Y | Y | Y | Y |
| **Singh et al., 2024** | Y | U | Y | U | Y | Y | Y | Y | Y | Y | Y | Y | Y |
| **Patel et al., 2012** | Y | Y | Y | Y | Y | N | Y | Y | Y | Y | Y | Y | Y |
| **Mohamed Saeed et al., 2017** | Y | N | Y | U | U | U | Y | Y | Y | Y | Y | Y | Y |
| **Verma et al., 2016** | Y | N | Y | N | U | U | Y | Y | Y | Y | Y | Y | Y |
| **Shoukheba and Ali, 2014** | Y | U | Y | N | U | U | Y | U | U | Y | Y | Y | Y |
| **Patel et al., 2025** | Y | U | Y | N | U | U | Y | U | U | Y | Y | Y | Y |
| **Vignesh et al., 2022** | Y | Y | Y | Y | Y | Y | Y | U | Y | Y | Y | Y | Y |
| **Nardi et al., 2020** | Y | Y | N | Y | Y | Y | Y | Y | Y | Y | Y | Y | Y |
| **Akash et al. 2021** | Y | U | Y | Y | U | U | Y | U | U | Y | Y | Y | Y |

Y: Yes, N: No, U: Unclear, N/A: Not applicable

B:

| **B: Quasi experimental study** | | | | | | | | | |
| --- | --- | --- | --- | --- | --- | --- | --- | --- | --- |
| **Study** | **Is it clear in the study what is the “cause” and what is the “effect” (i.e. there is no confusion about which variable comes first)?** | **Was there a control group?** | **Were participants included in any comparisons similar?** | **Were the participants included in any comparisons receiving similar treatment/care, other than the exposure or intervention of interest?** | **Were there multiple measurements of the outcome, both pre and post the intervention/exposure?** | **Were the outcomes of participants included in any comparisons measured in the same way?** | **Were outcomes measured in a reliable way?** | **Was follow-up complete and if not, were differences between groups in terms of their follow-up adequately described and analyzed?** | **Was appropriate statistical analysis used?** |
| **Al-Chalabi & Mohamed,**  **2019** | Y | Y | Y | Y | Y | Y | Y | Y | Y |
| **Aljaky et al., 2024** | Y | N | NA | NA | Y | Y | Y | Y | Y |
| **Sheikh et al., 2020** | Y | Y | Y | Y | Y | Y | Y | Y | Y |
| **Lauritano et al., 2023** | Y | N | NA | NA | Y | Y | U | Y | Y |

Y: Yes, N: No, U: Unclear, N/A: Not applicable

Table S3. Titles, Key Findings, and Reported Complications of the Included Studies

| **Title** | **Conclusion** | **Complications** |
| --- | --- | --- |
| Clinical and radiographic evaluation of non-surgical therapy with and without ozone gel application in controlled type 2 diabetic patients with periodontitis: a randomized controlled clinical trial | Ozone gel has been suggested as a promising natural adjunct therapy for diabetics to improve periodontal health. | No complications were reported. |
| Effect of ozone oil and non-surgical periodontal treatment in patients with type 2 diabetes. In-vivo and in-vitro studies with fibroblasts and Candida albicans | After interventions, no significant differences in periodontal indices were seen. However, both showed statistical differences between baseline and three months for GI, PPD, and CAL. | No complications were reported. |
| Ozone Gel in Chronic Periodontal Disease: A Randomized Clinical Trial on the Anti-Inflammatory Effects of Ozone Application | Ozonated gel was as effective as CHX gel. | No complications were reported. |
| Comparative Evaluation of the Efficacy of Subgingival Irrigation of Ozonated Olive Oil with Gingelly Oil in Chronic Periodontitis Patients | Clinical parameters such as PI and GI showed that ozonized oil is superior to gingelly oil in subgingival rinsing as an adjunct to SRP. | No complications were reported. |
| Clinical and Radiodensitometric Evaluation of Ozonated Olive Oil in the Management of Periodontal Condition in Smoker Patients | There was no significant difference between the Ozonated olive oil group and the Control group regarding the PI, GI, PPD and CAL. | NR |
| Effectiveness of the adjunctive use of ozone and chlorhexidine in patients with chronic periodontitis | The adjunctive use of ozonated olive oil significantly improves clinical and microbiological indices as effectively as CHX. | No complications were reported. |
| Efficacy of Olive Oil, Ozonated Olive Oil, and Chlorhexidine Gel as an Adjunct to Scaling and Root Planing in Chronic Periodontitis: A Clinical Study | Subgingival application of ozonated olive oil, olive oil, and CHX gel, along with SRP, significantly improved the outcome of periodontitis treatment. | No complications were reported. |
| Comparison of Ozonated Olive Oil and Chlorhexidine Gel as an Adjunct to Nonsurgical Periodontal Therapy for the Treatment of Chronic Periodontitis: A Randomized Controlled Clinical Trial | Ozonated olive oil could enhance the outcomes of SRP for the treatment of periodontal diseases. | No complications were reported. |
| Ozonized gels vs chlorhexidine in non-surgical periodontal treatment: A randomized clinical trial | Ozonized gel could be introduced as an alternative to CHX gel for treatment of periodontal diseases. | NR |
| Efficacy Ozone Therapy in Reducing Periodontal Disease | Treatment with adjunctive ozonated gel may not be associated with better outcomes. | NR |
| Effect of subgingival application of ozone oil versus olive oil as an adjunct to scaling and root planing in chronic periodontitis | Subgingival ozone oil application serves as an effective adjunctive therapy that contributes meaningfully to improving periodontal health. | No complications were reported. |
| Effect of ozonated oil versus chlorhexidine gel among Indian patients with chronic periodontitis | Ozonated oil, when used adjunctively with SRP, shows significant improvements in clinical parameters of chronic periodontitis comparable to Chlorhexidine gel. It demonstrates superior efficacy in reducing periodontal pocket depth and attachment loss compared to Chlorhexidine. | NR |
| Effect of subgingival application of topical ozonated olive oil in the treatment of chronic periodontitis: a randomized, controlled, double blind, clinical and microbiological study | The ozonated olive oil, adjunctive or mono-therapy, is efficient in improving periodontal indices. | No complications were reported. |
| Clinical and Microbiological Evaluation of Oleozone Gel in the Treatment of Chronic Periodontitis | SRP + ozonated olive oil gel group demonstrated superior outcomes in both clinical and microbiological evaluations. | No complications were reported. |
| Ozonized oral gel as an adjuvant in the treatment of periodontal disease: a preliminary report | This study showed the effectiveness of ozonized oil as a viable antimicrobial agent in periodontal disease treatment. | NR |
| The effects of subgingival application of ozonated olive oil gel in patient with localized aggressive periodontitis. A clinical and bacteriological study | They showed that ozonated olive oil gel could be a promising adjunct to SRP for aggressive periodontitis treatment. | No complications were reported. |
| Evaluating Ozonated Olive Oil and Chlorhexidine Gel as a Therapeutic Intervention for Periodontitis Stage II Grade A | Ozonated olive oil was as effective as CHX in enhancing SRP outcomes in periodontal disease as a valuable adjunct therapy. | NR |
| Effect of ozonated olive oil gel and chlorhexidine gel as an adjunct to scaling and root planing in chronic periodontitis – a clinical study | In patients with chronic periodontitis, CHX gel and ozonated oil showed similar therapeutic effectiveness. | No complications were reported. |
| Evaluation of Salivary Matrix Metalloproteinase (MMP-8) in Periodontal Patients Undergoing Non-Surgical Periodontal Therapy and Mouthwash Based on Ozonated Olive Oil: A Randomized Clinical Trial | SRP + ozonated olive oil mouthwash were found to be more effective on salivary MMP-8 reduction than SRP alone. | No complications were reported. |

***PPD***

**Ozone Therapy Without a Control Group**

Random-effect model

Ozone monotherapy

Fixed-effect model/ 12 weeks studies subgroup analysis

Leave-one out method

Funnel plot

Meta-regression

**Ozone Therapy Compared to a Control Group**

Random-effect model

Fixed-effect model/ 12 weeks studies subgroup analysis

Funnel plot

Funnel plot (Fixed-effect model/ 12 weeks studies subgroup analysis)

**Ozone Therapy Compared to CHX**

Random-effect model

Fixed-effect model

Fixed-effect model/ 12 weeks studies subgroup analysis

Leave-one out method

Funnel plot

Meta-regression (Meta-regression showed no significant effect of follow-up duration on PPD in the comparison between ozone therapy and CHX (coefficient = 0.0176, P = 0.774).)

*PI*

Subgroup analysis: Adjunctive therapy

Funnel plot (Subgroup analysis: Adjunctive therapy)

Meta-regression

**Ozone Therapy Compared to a Control Group**

Fixed-effect model

Random-effect model

Fixed-effect model/ 12 weeks analysis

Leave-one out model

Sensitivity analysis (Mohammad saeed et al. removing)

**Ozone Therapy Compared to CHX**

Fixed-effect model

Random-effect model

Subgroup analysis of 12 weeks

Subgroup analysis: Adjunctive therapy

Subgroup analysis: Monotherapy

Leave-one out method

Fixed-effect funnel plot

**Meta regression**

Meta-regression analysis showed no significant effect of follow-up duration (3 weeks to 3 months studies) on PPD (coefficient = 0.0275; P = 0.606), PI (coefficient = 0.0684; P = 0.351), or CAL (coefficient = -0.026; P = 0.897). However, additional data are needed to confirm this finding and strengthen the reliability of the analysis.
